# Computational Determination of Potential Multiprotein Targeting Natural Compounds for Rational Drug Design Against SARS-COV-2

**DOI:** 10.3390/molecules26030674

**Published:** 2021-01-28

**Authors:** Ziyad Tariq Muhseen, Alaa R. Hameed, Halah M. H. Al-Hasani, Sajjad Ahmad, Guanglin Li

**Affiliations:** 1 Key Laboratory of Ministry of Education for Medicinal Plant Resource and Natural Pharmaceutical Chemistry, Shaanxi Normal University, Xi’an 710062, China; ziyad.tariq82@gmail.com; 2 School of Life Sciences, Shaanxi Normal University, Xi’an 710062, China; 3 Department of Medical Laboratory Techniques, School of Life Sciences, Dijlah University College, Baghdad 00964, Iraq; alaa.raad@duc.edu.iq; 4 Department of Biotechnology, College of Science, University of Diyala, Baqubah 32001, Iraq; halahalhasani@sciences.uodiyala.edu.iq; 5 Foundation University Medical College, Foundation University Islamabad, Islamabad 44000, Pakistan; sajjademaan8@gmail.com

**Keywords:** SARS-CoV-2, COVID-19, multiprotein inhibiting natural compounds, virtual screening, MD simulation

## Abstract

SARS-CoV-2 caused the current COVID-19 pandemic and there is an urgent need to explore effective therapeutics that can inhibit enzymes that are imperative in virus reproduction. To this end, we computationally investigated the MPD3 phytochemical database along with the pool of reported natural antiviral compounds with potential to be used as anti-SARS-CoV-2. The docking results demonstrated glycyrrhizin followed by azadirachtanin, mycophenolic acid, kushenol-w and 6-azauridine, as potential candidates. Glycyrrhizin depicted very stable binding mode to the active pocket of the Mpro (binding energy, −8.7 kcal/mol), PLpro (binding energy, −7.9 kcal/mol), and Nucleocapsid (binding energy, −7.9 kcal/mol) enzymes. This compound showed binding with several key residues that are critical to natural substrate binding and functionality to all the receptors. To test docking prediction, the compound with each receptor was subjected to molecular dynamics simulation to characterize the molecule stability and decipher its possible mechanism of binding. Each complex concludes that the receptor dynamics are stable (Mpro (mean RMSD, 0.93 Å), PLpro (mean RMSD, 0.96 Å), and Nucleocapsid (mean RMSD, 3.48 Å)). Moreover, binding free energy analyses such as MMGB/PBSA and WaterSwap were run over selected trajectory snapshots to affirm intermolecular affinity in the complexes. Glycyrrhizin was rescored to form strong affinity complexes with the virus enzymes: Mpro (MMGBSA, −24.42 kcal/mol and MMPBSA, −10.80 kcal/mol), PLpro (MMGBSA, −48.69 kcal/mol and MMPBSA, −38.17 kcal/mol) and Nucleocapsid (MMGBSA, −30.05 kcal/mol and MMPBSA, −25.95 kcal/mol), were dominated mainly by vigorous van der Waals energy. Further affirmation was achieved by WaterSwap absolute binding free energy that concluded all the complexes in good equilibrium and stability (Mpro (mean, −22.44 kcal/mol), PLpro (mean, −25.46 kcal/mol), and Nucleocapsid (mean, −23.30 kcal/mol)). These promising findings substantially advance our understanding of how natural compounds could be shaped to counter SARS-CoV-2 infection.

## 1. Introduction

Coronaviruses (CoVs) cause infection of the upper respiratory tract in higher mammals and humans [1], and several outbreaks have been associated in the recent past with CoVs reported first time in the year 2002 as SARS, in 2012 as MERS, and in late 2019 as COVID-19 [2,3,4,5]. The recent pandemic of COVID-19 is caused by a relatively new strain named SARS-CoV-2 [6,7,8]. The virus origin is thought to be zoonotic, with potential of transmissibility between person-to-person, resulting in an exponential rise in the number of confirmed cases worldwide [9,10]. Through December 2020, more than 220 countries reported the virus, with more than 64 million individuals infected, and thousands are still getting infected each day. Approximately, the virus has a mortality rate between 5% to 10% [11,12]. Additionally, due to mandatory lockdowns, isolation, and quarantines, millions of lives have been disturbed. The pandemic also badly affected global health, society, and the economy, and these sectors are facing significant challenges [13]. Three vaccines (by Pfizer, Moderna, and AstraZeneca) are authorized by WHO for emergency use and are available to very limited populations. No specific anti-SARS-CoV-2 drugs are currently recommended for SARS-CoV-2 treatment, making the situation difficult to handle. Supportive therapeutics and preventative measures are being taken and are productive in managing the virus [14,15]. Various efforts to target critical proteins of SARS-CoV-2 pathogenesis, including Spike receptor-binding domain (RBD) [16,17,18], main protease (Mpro) [19], Nucleocapsid N terminal domain (NTD) [20], RNA-dependent RNA polymerase (RdRp) [21], papainlike protease (PLpro) [22], 2′-O-RiboseMethyltransferase [23], viral ion channel (E protein) [24], and angiotensin-converting-enzyme 2 receptor (ACE2) [25], are on the way. Targeting multiple pathogenesis specific proteins within a close network of interaction or dependent functionality would effectively propose effective drugs against the SARS-CoV-2 [26].

SARS-COV-2 Spike protein is key to the host cell infection pathway as it mediates ACE2 recognition, attachment, and fusion to the host cell [16]. The RBD of S1 subunit of the Spike trimer binds explicitly to the ACE2 receptor [27]. This RBD region is an attractive target for therapeutics as it contains conserved residues that are essential in binding to ACE2 [27]. The Mpro of coronaviruses has been studied thoroughly for drug making purposes. These are papainlike proteases involved in processing replicase enzymes [28]. It has 11 cleavage sites in 790 kD-long replicase lab polypeptide, demonstrating its prominent role in proteolytic processing [19,29]. High structural similarity and sequence identity are seen in Mpro from SARS-CoV-2 to that of the SARS-CoV Mpro. It comprises two catalytic domains: chymotrypsin and picornavirus 3C protease like domain. Each contains β-barrel that are six in number and are antiparallelly containing active diad H41 and C145 [30]. These proteases have emerged as essential drug targets as they have a crucial role in replication. Furthermore, inhibitors of Mpro are found to be significantly less cytotoxic as the protein share less similarity with human proteases [31]. Preliminary studies have suggested that HIV protease inhibitors, lopinavir/ritonavir, could be potentially used against SARS-CoV-2 [32]. Additionally, HIV protease inhibitor, Darunavir, and HCV protease inhibitor, Danoprevir, are under clinical studies and in vivo trials for the treatment of SARS-CoV-2 infection [33]. The PLpro enzyme is vital in processing the polypeptide to produce a functional replicase complex and aids in viral spreading [22]. PLpro also plays a role in evading host antiviral immune responses by cleaving proteinaceous modification on the host protein after the post-translation phase [34]. Thus, targeting this enzyme is useful in highlighting therapeutic strategies that can suppress the virus infection and prompt antiviral immunity. The N protein is significant in viral RNA replication and its packing into new virions, making this protein a good candidate for newer drug identification that is specific and biological active [20].

**Figure 1:**
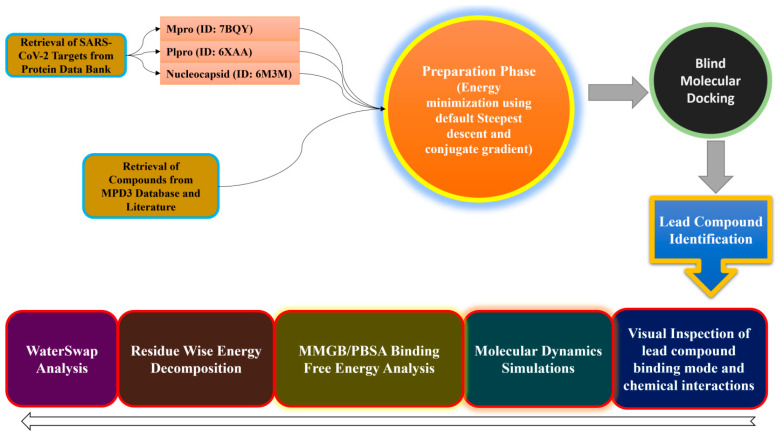
Schematic presentation of the methodology used in this current study.

In silico screening of drugs using different computer-aided drug designing applications greatly accelerate the rational drug design process. Ultimately, this saves time, and extra cost goes into the experimentation of leads that fail in the drug discovery process [35,36,37,38,39,40]. In this investigation, we performed a blind docking approach, followed by molecular dynamics (MD) simulation coupled with binding free energy techniques that dissect the structural dynamics and energy basis of molecular recognition [41,42]. The MPD3 phytochemical database [43] along with a pool of natural antiviral compounds were used against multiple SARS-CoV-2 protein targets to understand their binding mechanism and put forward a hypothesis on how to further optimize these structures to enhance selectivity and maximize anti-SARS-CoV-2 biological potency [44,45,46,47]. A schematic summary of the methodology used in this work is provided in Figure 1. The study results might have potential applications in designing new leads against SARS-CoV-2, which can target its multiple proteins as depicted in this study.

## 2. Results and Discussion

### 2.1. Molecular Docking

**Figure 2:**
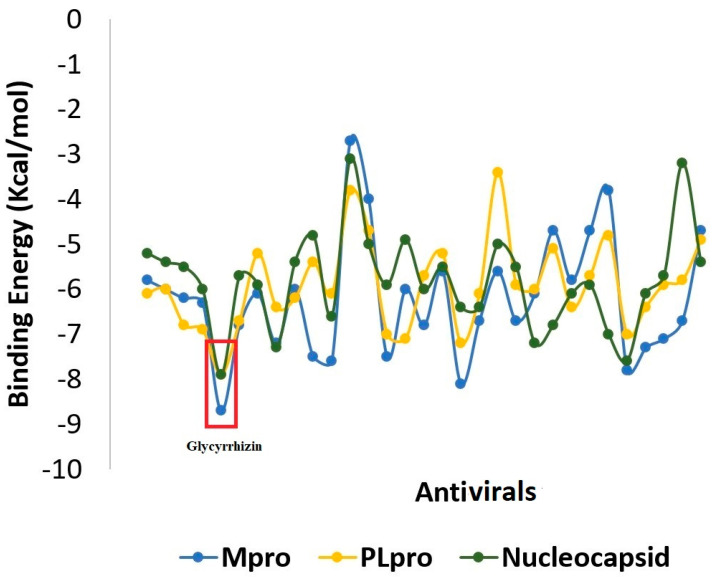
AutoDock binding affinity score of the compounds to the SARS-CoV-2 enzymes.

**Figure 3:**
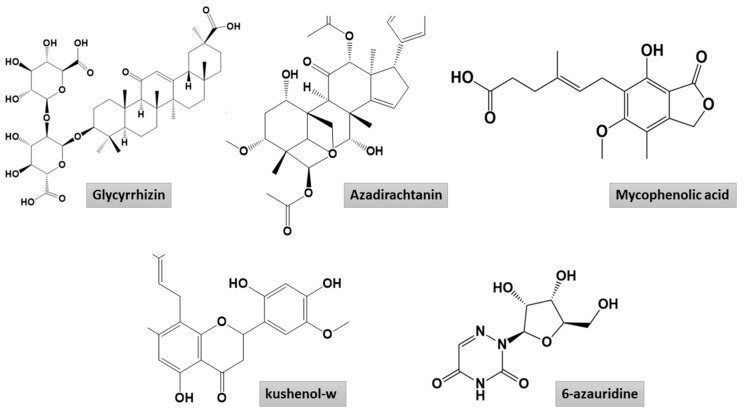
Two-dimensional presentation of high affinity binders to the SARS-CoV-2 proteins.

Molecular docking is a modeling approach investigating how the receptors and ligands fit together and how the enzymes interact with the ligands [48,49,50,51,52]. Docking calculations were performed in triplicate, and the compound conformations were ranked according to the binding energy in kcal/mol. We used remdesivir as control in docking. The compounds ranked consistently on top with the each receptor and showed a stronger binding score compared to remdesivir were selected for the downward analysis. A general overview of the binding energy of the compounds against the receptors used is presented in Figure 2. The top compound complex with each receptor was generated and subjected first to visual inspections to decipher atomic level interaction and determine the binding conformation. The docking analysis demonstrated glycyrrhizin followed by azadirachtanin, mycophenolic acid, kushenol-w, and 6-azauridine as the best binders among the ~5000 compounds used in this study. The 2D structures of these compounds are presented in Figure 3. Glycyrrhizin also showed stable interactions with the hotspot residues of SARS-CoV-2 spike protein receptor binding domain (RBD) in our previous study [53]. Glycyrrhizin-docked complex of each SARS-CoV-2 protein can be explained separately.

#### 2.1.1. Mpro–Glycyrrhizin Complex

**Figure 4:**
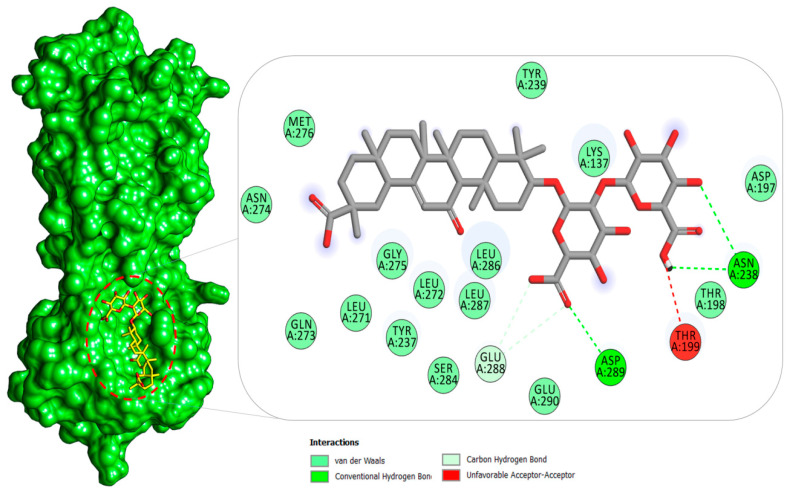
Binding pose of glycyrrhizin (in yellow stick) at the substrate binding pocket of Mpro (in the green surface). A 2D illustration of the glycyrrhizin chemical interactions at the docked site is also provided.

The Mpro of SARS-CoV-2 is a crucial enzyme and attractive drug target because of its central role in virus transcription and replication [54]. The docking study reported glycyrrhizin again as the best binder among the compounds used to the substrate-binding site of the Mpro (Figure 4). As seen in the binding with other receptors, the compound (2S,3S,4S,5R,6R)-6-(((2S,3R,4S,5S,6S)-6-carboxy-2,4,5-trihydroxytetrahydro-2H-pyran-3-yl)oxy)-3,4,5-trihydroxytetrahydro-2H-pyran-2-carboxylic acid was revealed to contribute in significant hydrogen bonding and other weak interaction at the active pocket of Mpro. At the binding cavity, the compound engages Asn238 through multiple hydrogen interactions, as well as Asp289. The rest of the compound structure makes a network of hydrophobic interactions mainly dominated by van der Waals contacts. To elucidate further the binding specificity and affinity of the glycyrrhizin for the active pocket residues of Mpro, the interaction profile was compared and contrasted with that for the reported cocrystallized N3 inhibitor [55]. Very low similarity in the binding interaction profile between the compounds was noticed; however, because of the difference in the compound structure, size, and preferred binding site, the pocket residues in contact with glycyrrhizin are close to the N3. This difference in the binding interaction points to the different glycyrrhizin-binding mechanism, where the active moiety favors binding with the P5 binding pocket that is absent in the case of the Mpro–N3 complex. The residues, particularly Asp197 and Thr198, flanked the active site, and any molecule involved in binding with these residues interfere with the natural substrate-binding, thus affecting the enzyme functionality [56]. Additionally, the bulk of the glycyrrhizin structure favors interactions with Domain II and Domain III of the Mpro, in addition to flanking residues of the substrate-binding pocket, thus possibly affecting the dimerization of Domain I and Domain II and rendering the enzyme noncatalytic [57]. Similarly, Zhang et al. reported Mpro complex with an α-ketoamide inhibitor. The cocrystalized lead identified binds to the same substrate binding site reported in this study [28]. Morever, calpain inhibitors and GC-376 analogs are also confirmed to accommodate in the same functional pocket [58]. Beside these, many in silico studies have demonstrated the binding affinity of drug molecules to this active side of Mpro [33,59,60,61].

#### 2.1.2. PLpro–Glycyrrhizin Complex

**Figure 5:**
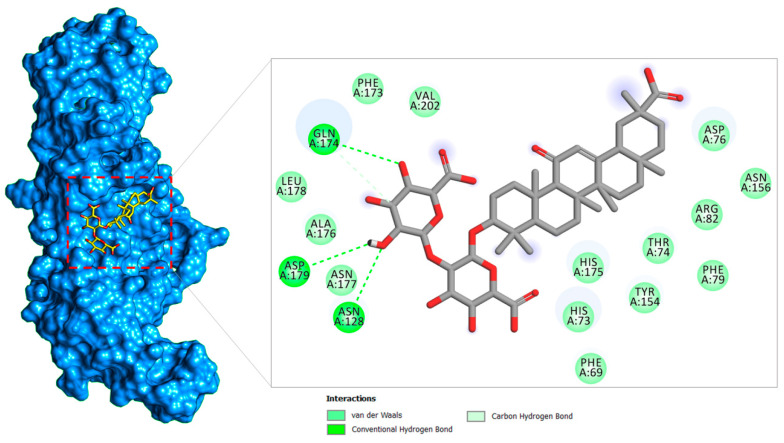
Binding pose of glycyrrhizin (in yellow stick) at the junction binding pocket of PLpro (in blue surface). A 2D illustration of the glycyrrhizin chemical interactions at the docked site is also provided.

The PLpro enzyme of SARS-CoV-2 is implicated in viral polyproteins processing that generate a replicase complex and assist in virus spreading. The enzyme also plays a fundamental role in cleaving post-translational proteinaceous modifications present on the host protein as a mechanism to avoid antiviral host immune responses [22]. The docked complex between PLpro and glycyrrhizin highlighted the compound binding at the central palm catalytic cavity (Figure 5). Good binding of the compound-rich electronegative oxygen in the (2*S*,3*S*,4*S*,5*R*,6*R*)-6-(((2*S*,3*R*,4*S*,5*S*,6*S*)-6-carboxy-2,4,5-trihydroxytetrahydro-2*H*-pyran-3-yl)oxy)-3,4,5-trihydroxytetrahydro-2*H*-pyran-2-carboxylic acid at the docked site is the output of several strong hydrogen bond interactions: Gln174, Asp179, and Asn128. Besides these residues, the compounds moiety also formed van der Waals interaction, critical from a stability perspective. The remainder of the compound structure produced van der Waals contacts at this central cavity. The preferred binding of glycyrrhizin is at the central palm, sandwiching the finger and thumb domains, adjacent to the active substrate-binding pocket, which makes a strong bond with many vital catalytic residues. In contrast to the cocrystallized peptide inhibitor VIR251, which has a different conformation and binds to a different substrate cavity site, the glycyrrhizin-binding site is close to the VIR251 site [62]. In terms of interacting binding residues, the glycyrrhizin correlates more with the GRL0617 inhibitor of SAR-CoV-2 PLpro [63]. Further, the effect of conformational change of the BL2 loop upon glycyrrhizin binding is important to evaluate in future studies to disclose the glycyrrhizin recognition mechanism.

In literature, many inhibitors of coronaviruses PLpro are documented that include zinc conjugate inhibitors, naphthalene, and thiopurine derivatives, and natural products [64]. These molecules are known to interact with the active site residues reported in this study. Tanshinones are reported to show inhibition of deubiquitinase and proteolytic activitiy of SARS-CoV PLpro [65]; 8-(Trifluoromethyl)-9*H*-purin-6-amine is a reversible noncovalent inhibitor, whereas N-Ethylmaleimide (NEM) modifies SARS-CoV PLpro Cys [63]. Moreover, 6-mercaptopurine (6MP) and 6-thioguanine (6TG) are slow and competitive inhibitors that form hydrogen bonds with catalytic residues of the SAR-CoV PLpro [66]. Several in silico studies also demonstrated a range of compounds that interfere with the functional site of SARS-CoV-2 PLpro [67,68,69,70].

#### 2.1.3. Nucleocapsid–Glycyrrhizin Complex

**Figure 6:**
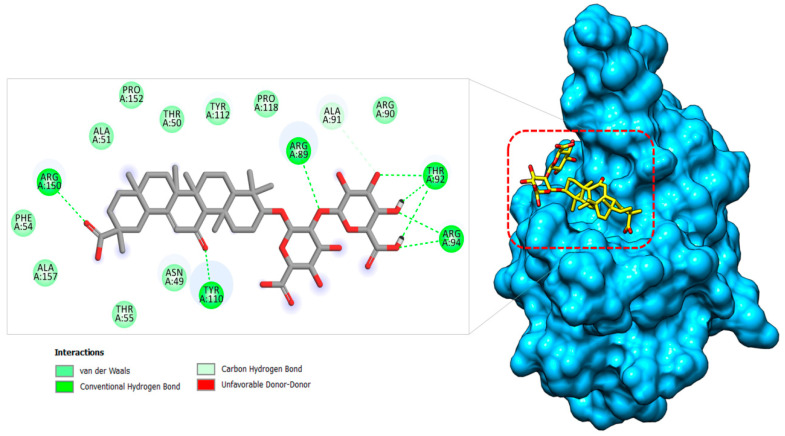
Binding pose of glycyrrhizin (in yellow stick) at the junction binding pocket of PLpro (in deep sky blue surface). A 2D illustration of the glycyrrhizin chemical interactions at the docked site is also provided.

The SARS-CoV-2 N protein is an RNA binding protein and offers several functions of viral transcription and replication [20]. It particularly plays a pivotal role in helical ribonucleoprotein packing during RNA genome packing, regulating RNA replication, and modulating infected cell metabolism. Blocking of this protein could lead to blocking viral replication, and thus an attractive target for drug development. The compound glycyrrhizin was found to prefer docking at the loop region 1 at the junction between the β-sheet core and β-hairpin (Figure 6). The molecule is aligned perfectly along the cavity volume where its (*2S,3S,4S,5R,6R*)-6-(((*2S,3R,4S,5S,6S*)-6-carboxy-2,4,5-trihydroxytetrahydro-2*H*-pyran-3-yl)oxy)-3,4,5-trihydroxytetrahydro-2*H*-pyran-2-carboxylic acid part is connected to the β3 and β4 sheets of the β-hairpin. Here, this chemical moiety is involved in hydrogen bonding with Thr92, Arg94, and Arg89, and van der Waals contact with Arg90 and Ala91. The (2S,4aS,6aS,6bR,8aS,12aS,12bR,14bR)-2,4a,6a,6b,9,9,12a-heptamethyl-13-oxo-1,2,3,4,4a,5,6,6a,6b,7,8,8a,9,10,11,12,12a,12b,13,14b-icosahydropicene-2-carboxylic acid region of the compound produced hydrogen bonding with residues (Tyr110 and Arg150) and van der Waal contacts with residues (Asn49,Thr50, Als51, Phe54, Thr55, Tyr112, Pro118, Pro152, and Ala157) of β1, β2, β4, β5, β6, and β7 of the β-sheet core of the protein. Bhowmik et al. reported strong binding of Rutin, Doxycycline, Caffeic acid, Ferulic acid, Simeprevir, and Grazoprevir with several functional residues of the SARS-CoV-2 nucleocapsid protein reported in this study [71].

### 2.2. MD Simulation Analysis

In computer-aided drug design, MD simulations are essential in providing detailed biomolecule dynamical structural information and surface wealth of protein–ligand interactions, energetic data that are foremost to understanding the structural–functionality relationship of target protein principle in ligand recognition/interactions [37,72,73]. This set of information has tremendous applications in guiding novel drug design, thereby making MD simulation a successful tool in the modern drug discovery framework.

#### 2.2.1. Root-Mean-Square Deviation (RMSD) Analysis

**Figure 7:**
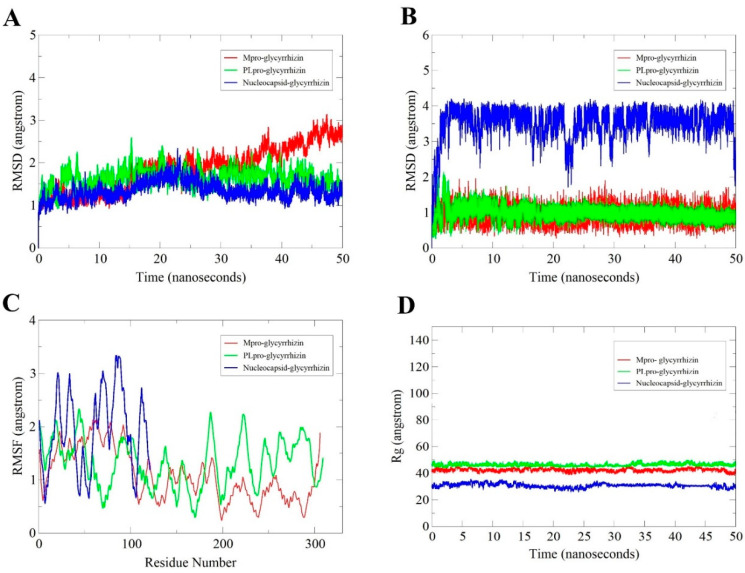
Statistical parameters calculated based on simulation trajectories. Receptor RMSD plots (**A**), glycyrrhizin RMSD plots (**B**), receptor RMSF (**C**), and receptor Rg (**D**).

MD simulation of 50 ns was performed for each receptor with bound glycyrrhizin to elucidate the compound binding stability and extract receptors/compound structural information that is key in the binding that may be altered to iMprove binding conformation and, ultimately, compound affinity for the target biomolecules. First, RMSD of receptors in each complex was estimated as carbon alpha deviations by superimposing 50,000 snapshots over the initial reference structure versus time (Figure 7A). RMSDs of all three complexes were found: Mpro (maximum, 3.14 Å; mean, 1.97 Å), PLpro (maximum, 2.59 Å; mean, 1.64 Å), and Nucleocapsid (maximum, 2.34 Å; mean, 1.32 Å). All of the receptors are relatively stable in terms of 3D structure, and no flexibility in secondary structures was noticed. As a consequence, glycyrrhizin binding pose was not altered, thus reflecting strong and stable complex formation.

#### 2.2.2. Glycyrrhizin Conformation Stability

**Figure 8:**
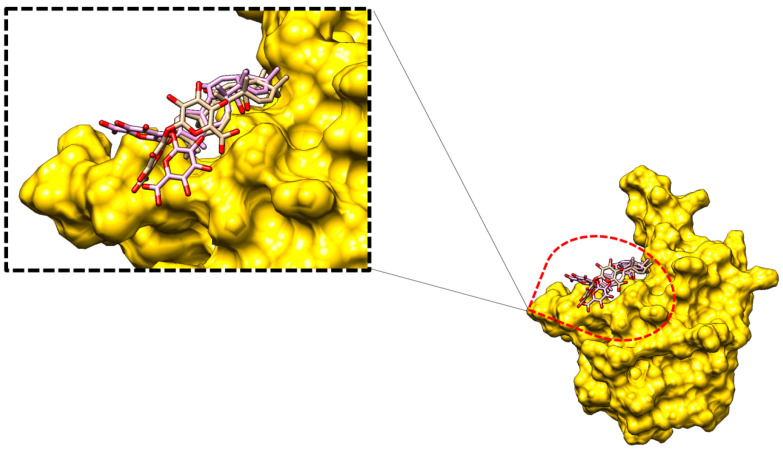
Binding mode dynamics of glycyrrhizin in the MD simulation at the initial time (in tan stick) versus at the end time (in plum stick).

In addition, the MD simulation trajectories were examined to disclose information about the glycyrrhizin conformation stability with the receptors (Figure 7B). The glycyrrhizin RMSD with the receptors is Mpro (maximum, 2.56 Å; mean, 0.93 Å), PLpro (maximum, 2.14 Å; mean, 0.96 Å), and Nucleocapsid (maximum, 4.20 Å; mean, 3.48 Å). The molecules disclosed high stable, except for some deviations in the glycyrrhizin binding mode with the Nucleocapsid protein; therefore, the end MD simulation snapshot over the initial was superimposed to understand the compound dynamics. The (*2S,3S,4S,5R,6R*)-6-(((*2S,3R,4S,5S,6S*)-6-carboxy-2,4,5-trihydroxytetrahydro-2*H*-pyran-3-yl)oxy)-3,4,5-trihydroxytetrahydro-2*H*-pyran-2-carboxylic acid fragment of the glycyrrhizin is flexible in an attempt to establish a more stable conformation. This moiety left its original site of interaction and moved more towards the β-core sheet for binding (Figure 8).

#### 2.2.3. Root-Mean-Square Fluctuation (RMSF) Analysis

The residual flexibility and stability of the receptors in the presence of glycyrrhizin were further elucidated (Figure 7C). Mean RMSF for Mpro is 1.4 Å, PLpro is 1.57 Å, and Nucleocapsid is 1.9 Å. These values suggest good agreement on intermolecular stability.

#### 2.2.4. Radius of Gyration (Rg) Analysis

Additionally, Rg analysis was performed to evaluate protein compactness and structural equilibrium over the simulation time (Figure 7D). The Rg of the systems follows: Mpro–glycyrrhizin (45.62 Å and 42.28 Å), PLpro–glycyrrhizin (50.29 Å and 46.23 Å), and Nucleocapsid–glycyrrhizin (35.71 Å and 30.70 Å). All three systems are quite stable and remain compact.

### 2.3. MMGB/PBSA Analysis

To get a deeper insight into the compounds binding potential with the SARS-CoV-2 enzymes used, binding free energies were estimated using MMGBSA and MMPBSA techniques. Additionally, per residue decomposition assay was accomplished to highlight residues that contribute majorly to the compound’s stability at the docked position and, ultimately, to the strong intermolecular interactions. To this objective, 100 frames were picked at time intervals of 50 ps from the simulation trajectories, discarding the water molecules and counterions. Detailed binding energies of the complexes are listed in Table 1 All of the binding interactions are energetically favorable, resulting in the formation of stable complexes. In all of the complexes, gas-phase energy dominates the system energy with significant contribution from van der Waals compared to electrostatic energy’s minor role. The polar solvation energy is illustrated to play a nonfavorable part in binding, whereas the nonpolar energy seems to be vital in complex equilibration. The MMGBSA net binding-energy-ranked stability of the complexes follows: PLpro–glycyrrhizin > Spike–glycyrrhizin > Nucleocapsid–glycyrrhizin > Mpro–glycyrrhizin. The MMPBSA ranking follows: PLpro–glycyrrhizin > Spike–glycyrrhizin > Mpro–glycyrrhizin > *N*–glycyrrhizin.

### 2.4. Per-Residue Decomposition

The atomic-level contribution of each residue from the enzymes to the compound binding was elucidated further. Those with an average binding energy of <1 kcal/mol were categorized as hotspot residues because of their significant overall complex stability contribution [74,75]. In the case of Mpro–glycyrrhizin interaction, Asn238 and Asp289 are vital in holding the compound at the docked site. Phe69, Asn128, Gln174, and Asp179 residues are critical in bridging PLpro enzyme with glycyrrhizin compound. The primary hotspot residues in Nucleocapsid–glycyrrhizin complex are Thr92, Arg94, Tyr110, and Arg150. It was further noticed that the van der Waals energy, as noted earlier, dominates the overall binding interaction energy. Hotspot residues of each receptor that are in direct contact and key in the stabilization of glycyrrhizin are presented in Table 2.

### 2.5. WaterSwap Binding Energy

WaterSwap uses an explicit solvation system that considers interaction details of protein–water, protein–water–ligand, and ligand–water. Such information is not provided in the MMGB/PBSA; therefore, it is not reliable for predicting the role of water molecules in biomolecule–ligand interactions [76]. Specifically, this holds great importance in an instance where the ligand is bridged to the receptor through water molecules. The WaterSwap method has been successfully applied to various biological systems and proved critical in determining absolute binding free energy. For each complex, the WaterSwap energies converged significantly after running 1000 frames. All the values also concluded good stability of intermolecular docked conformation. WaterSwap energies for each complex are shown in Table 3.

## 3. Materials and Methods

### 3.1. Target Proteins Preparation

The anti-SARS-CoV-2 targets (Mpro PDB code: 7BQY, PLpro PDB code: 6XAA, and Nucleocapsid PDB code: 6M3M) were retrieved and prepared using the AMBER18 program [77]. Ff14SB force field [78] was used for amino acid parameterization. To add complementary hydrogen atoms missed by the crystallography, the tleap module of AmberTools18 was employed. Energy minimization of the targeted proteins was done first for 1000 steepest descent steps, and then by 500 conjugate gradient steps, allowing the step size to be 0.02 Å. Charge addition was done through the Gasteiger method.

### 3.2. Compound Preparation

The MPD3 phytochemical database (https://www.bioinformation.info/), in addition to reported natural antiviral compounds, were used in this study to filter molecules that show best binding affinity to the selected SARS-CoV-2 multiple targets. The library containing ~5000 natural compounds was imported to PyRx 0.8 software [79], where they were minimized for optimal energy and followed by conversion to pdbqt format for use in virtual screening against the mentioned targets.

### 3.3. Structure-Based Virtual Screening

Virtual screening of the compounds against of the targets used was done using the AutoDock Vina in PyRx [80] on Windows 10-supported Dell system (processor: Intel(R) Core(TM) i7-8550U CPU @ 1.80 GHz with a 64-bit operating system, ×64-based processor, a memory of 8.00 GB). First, the docking protocol was validated by docking cocrystallized ligands to the protein keeping the docking parameters default except for the sphere around the binding site, which was set to 15 Å. Validation was also done by comparing the best-ranked compounds conformation relative to the crystallized ligand by root-mean-square deviation (RMSD) [81]. Docking of the compound to the targets was accomplished by using the same set of parameters described for the validation procedure and run in triplicates to absolute consistency of the results. The docked solutions were clustered, considering an RMSD value of 1 Å. The binding mode of compounds with the lowest binding energy in kcal/mol was refined in MD simulations.

### 3.4. MD Simulations

MD simulations of the docked solutions were performed using AMBER18 [77]. Each top complex was explicitly solvated with water molecules, and then to get a neutral system, counter ions were added. Afterward, using the TIP3P solvent model, a water box of thickness 12 Å was created to surround the complex [82]. Simulation of the complex was done through periodic boundary conditions where electrostatic interactions were modeled with the particle–mesh Ewald procedure [74]. In the process, a threshold value of 8 Å was defined for nonbounded interactions. Water molecules were minimized for 500 cycles, followed by complete system minimization for 1000 rounds. Then, each system temperature was gradually scaled to 300 K. Equilibration of the systems was achieved under the NPT ensemble for 100 ps. This involves equilibration of both counter ions and water molecules while considering restraint on solutes in the first phase for 50 ps; subsequent protein side chains were relaxed. MD simulation of 50 ns was performed at 300 K and 1 atm for two fs under the NPT ensemble. Hydrogen and covalent bonds were constrained using the SHAKE algorithm [83], whereas system temperature was controlled through Langevin dynamics [84]. The initial structure was used as a reference, and CPPTRAJ [85] of AMBER was run to generate a root-mean-square deviation (RMSD) plot to check the system MD simulation convergence [81]. Ligand structural flexibilities were calculated by ligand RMSD. Furthermore, hydrogen bond analysis was performed to investigate hydrogen bonds formed between the compounds and amino acids present within the docked site vicinity.

### 3.5. MMGB/PBSA Analysis

The binding free energy (ΔG binding) of the complexes was estimated using the AMBER18 MM/PBSA method [42,86]. One hundred snapshots were considered from simulation trajectories at a regular time interval to calculate the free energy difference.

ΔG_binding_ = G_complex_ − (G_protein_ + G_ligand_)

ΔG = ΔG_gas_ + ΔG_solv_ − TΔS

ΔG_gas_ = Δele + ΔGvdw

ΔGsolv = ΔG_GB_ + ΔG_SA_

ΔG_SA_ = **γ** × SASA × b

In these equations, G_complex_ is delta free energy of the complex, G_protein_ is delta free energy of the protein, and G_ligand_ is delta free energy of the ligand; ΔG_gas_ represents gas-phase energy and can be split into delta electrostatic (ΔE_ele_), and delta van der Waals (ΔE_vdw_) energy; and the ΔG_solv_ term stands for solvation free energy, which comprises polar (ΔG_GB_) and nonpolar (ΔG_SA_) energy. In the ΔG_GB_, the εw value is set to 80, and εp is selected as 1.0. Linear combinations of the pairwise overlap method are used to estimate the solvent-accessible surface area (SASA).

### 3.6. WaterSwap Analysis

WaterSwap [76,87] was additionally done over the last 10 ns of MD simulation for a total of default 1000 iterations, keeping the sample size of Monte Carlo simulation to 1.6 × 10^9^. The absolute binding energy of each complex was estimated using three useful algorithms: thermodynamics integration, free energy perturbation, and Bennett’s. The energy value <1 kcal/mol represents a good convergence of the system [75].

## 4. Conclusions

In this study, we found glycyrrhizin as the most significant natural compound that can act as a double-edged sword and inhibit multiple proteins of SARS-CoV-2. This compound has a high binding affinity for all of the SARS-CoV-2 receptors used in this study and had a stable binding mode in the MD simulation time. The compound revealed important interactions with all receptors, and thus requires further consideration in future anti-SARS-CoV-2 therapeutic studies. Glycyrrhizin has been previously documented to have therapeutic applications against SARS-CoV, chronic hepatitis C, and HIV-1 [88]. The molecule is clinically useful and had few toxic reactions. One way to overcome toxicity is by allowing low concentration of the drug in the cells (<100 µg/mL) [89]. Glycyrrhizin has been reported to inhibit viral penetration and effective both during the viral infection and postinfection [90]. It was previously demonstrated that the glycyrrhizin binds with good affinity to the human ACE2 and interacts with Asp30, Gln288, Arg393, and Arg559 residues, hence also underlines its potential to target the SARS-CoV-2 Spike protein RBD attachment to the human ACE2 receptor [90]. It also was shown that glycyrrhizin can be employed in synergism along with other plant-based molecules to treat SARS-CoVs [91]. From a pharmacological perspective, the glycyrrhizin prevents the production of intracellular reactive oxygen species, activates interferon production, downregulates proinflammatory cytokines, lowers airway exudate production, and inhibits thrombin [45,92]. The compound was also computationally characterized previously to bind with good affinity to SARS-CoV-2 main protease [93]. Therefore, additional structural modification to lower the side effects and enhance the clinical efficacy of this compound is of high interest to treat SARS-related infections.

## Figures and Tables

**Table 1:** Binding free energy components of SARS-CoV-2 enzyme complexes with glycyrrhizin. The energy values are provided in units of kcal/mol.

Method	Energy Component	Mpro–Glycyrrhizin	PLpro–Glycyrrhizin	Nucleocapsid–Glycyrrhizin
MMGBSA	Van der Waals Energy	−36.50	−61.10	−37.97
	Electrostatic Energy	−13.92	−8.53	8.75
	Polar Solvation Energy	30.19	26.79	3.15
	Nonpolar Solvation Energy	−4.19	−5.85	−3.97
	Gas Phase Energy	−50.42	−69.63	−29.22
	Solvation Energy	25.99	20.93	−0.82
	Total Binding Energy	−24.42	−48.69	−30.05
MMPBSA	Van der Waals Energy	−36.50	−61.10	−37.97
	Electrostatic Energy	−13.92	−8.53	8.75
	Polar Solvation Energy	42.56	35.77	6.65
	Nonpolar Solvation Energy	−2.94	−4.31	−3.38
	Gas Phase Energy	−50.42	−69.63	−29.22
	Solvation Energy	39.62	31.46	3.27
	Total Binding Energy	−10.80	−38.17	−25.95

**Table 2:** Hotspot residues identified that played a significant role in interaction with the glycyrrhizin.

Complex	Residues	MMGBSA	MMPBSA
Mpro–Glycyrrhizin	Lys137	−1.74	−1.51
	Asp197	−1.76	−0.45
	Thr198	−1.50	−1.76
	Thr199	−1.18	−2.84
	Tyr237	−1.46	−1.89
	Asn238	−2.98	−3.45
	Tyr239	−1.54	−1.48
	Leu271	−1.24	−1.69
	Leu272	−1.65	−3.48
	Gln273	−1.14	−1.24
	Asn274	−1.56	−1.42
	Met276	–1.73	−1.98
	Ser284	−1.89	−1.51
	Leu286	−1.98	−1.47
	Leu287	−1.48	−2.84
	Glu288	−1.44	−1.84
	Asp289	−3.74	−3.54
	Glu290	−1.88	−5.45
PLpro–Glycyrrhizin	Phe69	−2.54	−3.54
	His73	−2.11	−2.45
	Thr74	−1.82	−1.45
	Asp76	−1.99	−1.68
	Phe79	−1.47	−1.46
	Arg82	−1.82	−1.12
	Asn128	–4.41	−1.39
	Tyr154	−1.61	−1.48
	Asn156	−1.61	−5.24
	Phe173	−1.11	−1.58
	Gln174	−5.48	−3.61
	His175	−1.94	−1.48
	Ala176	−1.69	−1.12
	Asn177	−1.81	−1.62
	Leu178	−1.64	−1.11
	Asp179	−2.47	−1.83
	Val202	−1.43	−1.19
Nucleocapsid–Glycyrrhizin	Asn49	−1.99	−2.54
	Thr50	−1.81	−1.42
	Ala51	−1.25	−2.45
	Phe54	−1.66	−3.15
	Thr55	−1.65	−1.12
	Arg89	−2.74	−1.84
	Thr92	−2.45	−3.65
	Arg94	−4.66	−2.48
	Tyr112	−1.45	−1.24
	Tyr110	−3.74	−3.51
	Pro118	−1.89	−1.48
	Arg150	−2.78	−3.58

**Table 3:** WaterSwap absolute binding energy estimation for all four complexes.

Algorithm	Mpro–Glycyrrhizin	PLpro–Glycyrrhizin	Nucleocapsid–Glycyrrhizin
Bennett’s	−22.39	−25.84	−22.34
Free energy perturbation	−22.48	−25.94	−23.83
Thermodynamic integration	−22.47	−24.61	−23.45
Mean	−22.44	−25.46	−23.30

## Data Availability

The data presented in this study are available within the article.

## Sample Availability

Not available.
